# Mechanism of improving quality of dry-aged pork loins in scoria-containing *onggi*, Korean earthenware as a storage container

**DOI:** 10.5713/ab.22.0438

**Published:** 2023-01-11

**Authors:** Sung-Su Kim, Dong-Jin Shin, Dong-Gyun Yim, Hye-Jin Kim, Doo Yeon Jung, Hyun-Jun Kim, Cheorun Jo

**Affiliations:** 1 Department of Agricultural Biotechnology, Center for Food and Bioconvergence, and Research Institute of Agriculture and Life Science, Seoul National University, Seoul 08826, Korea; 2 Department of Applied Animal Science, Kangwon National University, Chuncheon 24341, Korea; 3 Institute of Green Bio Science and Technology, Seoul National University, Pyeongchang 25354, Korea

**Keywords:** Lactic Acid Bacteria, Meat Aging, *Onggi*, Permeability, Pork Loin, Scoria

## Abstract

**Objective:**

Many scientists have investigated solutions to reduce microbiological risks in dry-aged meat after the dry-aging technology was revived for high quality and value-added premium meat product in the market. This study aimed to investigate the effect of scoria powder in *onggi* (Korean earthenware) on the meat quality of pork loins during 21 days of dry aging and to elucidate its mechanism of action.

**Methods:**

The pork loins were randomly divided into three groups: aged in vacuum-packaging, *onggi* containing red clay only (OR), and *onggi* containing 30% red clay and 70% scoria powder (OS). Microbial analyses (total plate count and *Lactobacillus* spp.) and physicochemical analyses (pH, shear force, volatile basic nitrogen [VBN], water activity, 2-thiobarbituric acid reactive substances, water content, water holding capacity, cooking loss, and color analysis) of aged meat were conducted. Far-infrared ray emission, quantification of immobilized *L. sakei* and microstructure of *onggi* were investigated to understand the mechanism.

**Results:**

On day 21, the meat aged in OS exhibited lower pH, shear force, VBN, and water activity than those aged in OR, along with an increase in the number of *Lactobacillus* spp. OS had a smaller pore diameter than OR, implying lower gas permeability, which could promote the growth of *L. sakei*.

**Conclusion:**

OS improved the microbiological safety and storage stability of pork loin during dry aging by increasing number of *Lactobacillus* spp. possibly due to low permeability of OS.

## INTRODUCTION

Aging enhances the tenderness, flavor, and overall acceptance of meat [1]; it induces meat tenderization by its inherent proteolytic enzymes (e.g., cathepsins, proteasomes, and calpains) and increases the formation of flavor compounds. The aging methods are primarily sorted into wet and dry aging, depending on the use of vacuum packaging (VP) [2].

Wet aging is a method that uses VP at designated temperatures [1]. Vacuum packaging limits the proliferation of aerobic bacteria in wet-aged meat, but the flavor of which does not change during the aging period [2]. Dry aging is a preservation method in air under controlled relative humidity, air velocity, and temperature, without VP [1]. Dry-aged meat is recognized as a premium product because of its characteristic beefy and roasted flavors [2]. However, dry-aged meat is exposed to microbiological risks by fluctuating temperature and humidity and is in constant risk of cross-contamination as it is exposed to the air [2]. To commercialize dry-aged meat with a highly acceptable flavor and safety, different dry-aging methods have been developed and applied in the industry. However, it is still difficult to confirm its effect and availability.

Korean earthenware (*onggi*) has been utilized to store and ripen foodstuffs such as cereals and vegetables [3]. Fermentation with *onggi* induced better product quality of food compared to that without it [4]. It is known that barrier properties against moisture and gas determine food quality in food preservation in *onggi*, relying on interactions between the inner and external environments [3]. Several studies [3,4] showed that *onggi* with controlled permeability could create a modified atmosphere for ripening products inside it, and the barrier traits of *onggi*, which are related to gas and moisture permeation, can be controlled and diversified using different clay formulations.

Scoria, which is widely distributed over the parasitic volcano area on Jeju Island, is an alkalescent and volcanogenous soil composed of rocks, sand, and ash that formed during the eruption of a volcano. The main ingredients of scoria are SiO_2_ (30% to 50%), Al_2_O_3_ (10% to 20%), and F_2_O_3_ (10% to 20%), which impart its own physical properties, such as high far-infrared ray (FIR) irradiation and antibacterial activity [5,6]. As such, scoria has been used because of its high FIR-radiating properties [7,8]. Indeed, Lin [9] stated that wavelength from FIR irradiation cause vibration, which could deform the protein and nucleic acid of bacteria to prevent meat spoilage.

Some studies have introduced the effects of *onggi* with different clay ratios on the foods stored within it [3,4]. However, no data are available regarding the effects of scoria-containing *onggi* on meat quality during dry aging or its mechanism of action. Therefore, the present study aimed to investigate the effect of scoria powder in *onggi* on meat quality and microbiological properties of pork loins for 21 days of dry aging and to elucidate the mechanism involved.

## MATERIALS AND METHODS

### Preparation of *onggi*

Two types of *onggi* were used in the current study; one was an *onggi* consisting of 100% red clay (OR), and the other was composed of 70% scoria powder and 30% red clay (OS). Pugging was made from each *onggi* soil and formed into 24 L cylindrical jars, with a height of 37 cm and a 1.7 cm thickness. After drying for 24 h in the shade, they were fired at 750°C for approximately 7 h in an electric kiln (SI 700; Seil Inc., Ulsan, Korea), followed by natural cooling. The vessels were then coated with glazing on the exterior and fired in a kiln with a gradually increasing temperature at 5°C/min to 450°C and 1,100°C for 5.5 h. Lastly, the vessels were heated at 1,190°C for 20 min, followed by slow cooling. All *onggi*es were washed with tap water and dried under the sunlight for 4 hours, followed by being sterilized by 70% ethanol before use.

### Raw material and aging process

**Figure 1:**
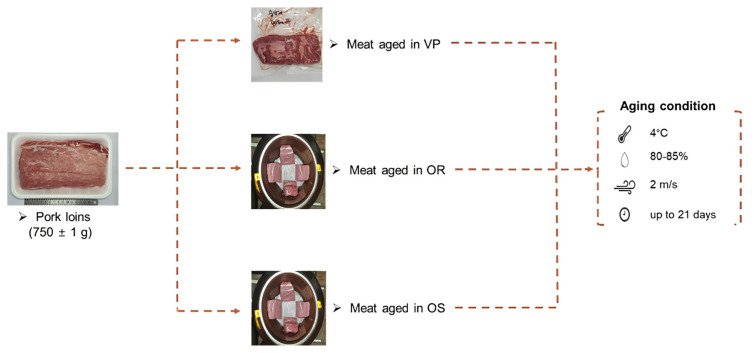
Graphical illustration of the experimental design. VP/OR/OS refers to vacuum-packaging (VP), *onggi* containing red clay only (OR), and *onggi* containing 30% red clay and 70% scoria powder (OS).

Figure 1 shows the overall experimental design and conditions. At 24 h postmortem, pork loins were obtained from a local slaughterhouse (Seoul, Korea). Thirty samples (750±10 g) were randomly assigned to three groups (n = 3 per group): meat aged in VP, OR, and OS, respectively. All were allocated to each of the four aging periods (0, 7, 14, and 21 days) in a walk-in cooler under specific conditions (temperature, 4°C; relative humidity, 80% to 85%; and air velocity, 2 m/s). Loins for VP were vacuum-packaged (HFV-600V; Hankook Fujee Co., Ltd., Siheung, Korea) in low-density polyethylene/nylon bags (O_2_ permeability of 22.5 mL/m^2^/24 h atm at 60% RH/25°C and water vapor permeability of 4.7 g/m^2^/24 h at 100% RH/25°C). Three ORs and OSs were used for aging, and loins for OR and OS were placed in each *onggi*.

After aging was completed, meat blocks were transferred to a clean bench of laboratory by aseptic containers. The samples for microbes were taken after removing the crust on the surface of meat in the clean bench. Most analyses were evaluated immediately when the samples were fresh, except for volatile basic nitrogen (VBN) and 2-thiobarbituric acid reactive substances (TBARS). Samples for VBN and TBARS were stored in vacuum-packaged conditions at −70°C until further analyses.

### Microbial growth

Samples (25 g) in each ripening condition were blended with 225 mL 0.85% saline solution for 2 min using a stomacher (BagMixer 400; Interscience Ind., Paris, France). One milliliter of each sample dilution was poured into agar. The experiment was carried out in a clean bench. Total plate count (TPC) and *Lactobacillus* spp. were enumerated using plate count agar (Difco Laboratories, Detroit, MI, USA) and *lactobacilli* MRS broth with agar (Difco Laboratories, USA), respectively. The agar plates for TPC and *Lactobacillus* spp. were incubated at 37°C for 72 and 96 h, respectively, and the number of colonies was counted (log CFU/g).

### Microbial isolation and identification

*Lactobacillus* spp. on *lactobacilli* MRS broth with agar at days 14 and 21 were used for microbial isolation, and colonies were dissociated into different plates depending on their phenotypic characteristics, such as shape, size, and color. The colonies that were separated into different plates were used for polymerase chain reaction analysis.

Colonies on each plate were identified using 16s rRNA sequencing. RNA was extracted and amplified as follows: i) initial denaturation at 95°C for 5 min; ii) denaturation at 95°C for 0.5 min, iii) annealing at 55°C for 2 min; iv) extension at 68°C for 1.5 min for 30 cycles; and v) final extension at 68°C for 10 min. Identification was performed using the RNA database available at the National Center for Biotechnology Information.

### pH and volatile basic nitrogen

Meat samples (1 g) were homogenized with Deionized distilled water (DDW, 9 mL), and the pH was measured using a pH meter (Seven2Go; Mettler-Toledo, Schwerzenbach, Switzerland) following a method [2]. Prior to the measurement, the pH meter was calibrated with standard buffers at 4.01, 7.00, and 9.21 (Mettler-Toledo Inc., Switzerland).

VBN was analyzed using the method described by Lee et al [10]. Each sample (3 g) in 27 mL of DDW was homogenized for 30 s (T25 digital ULTRA TURRAX; IKA, Staufen, Germany) followed by centrifugation (Continent 512R; Hanil Co., Ltd., Incheon, Korea) at 2,265×g for 10 min. After filtration, 0.01 N boric acid and indicator solution (0.66% methyl red in ethanol:0.66% bromocresol green in ethanol = 1:1 [v/v]) were placed individually in the inner section of a conway (Sibata Ltd., Sitama, Japan). Then, 1 mL of sample and 50% potassium carbonate were added into the outer section of the conway, after which the lid was sealed immediately. The conways were incubated at 37°C for 1 h and titrated with 0.01 N hydrogen chloride. The VBN values were expressed in mg/100 g.

### 2-Thiobarbituric acid reactive substances

TBARS was analyzed using the method described by Lee [10]. Samples (5 g) were homogenized in 15 mL of DDW with 50 μL of butylated hydroxytoluene in ethanol at 9,600 rpm for 30 s (IKA, Germany), followed by centrifugation at 4°C and 2,265×*g* for 15 min (Hanil Co., Ltd., Korea). The supernatant was filtered through No.4 filter paper (Whatman No. 4; Whatman PLC., Middlesex, UK), and a thiobarbituric acid solution in trichloroacetic acid was added. Following vortexing, the mixtures were heated at 90°C for 30 min and then centrifuged again at 4°C and 2,265×*g* for 15 min. The absorbance of the supernatant was measured at 532 nm using a spectrophotometer (Otizen 2120 UV plus; Mecasys Co. Ltd., Daejeon, Korea). The results are expressed as mg malondialdehyde (MDA)/kg meat.

### Shear force, water activity, water holding capacity, water content, and cooking loss

Shear force was analyzed the method explained by Kim et al [11]. The samples were cut parallel to the muscle fiber into rounded cores (1.27 cm diameter), and placed under a Warner-Bratzler shear probe, perpendicularly to the muscle fiber. The water activity (a_w_) was measured using a Rotronic HygroPalm HP23-Aw-A meter (Rotronic AG, Bassersdorf, Switzerland). The water holding capacity (WHC) of ground pork loin was calculated by following formula (1). The water content was determined using related-formula (2). The cooking loss of the meat blocks (100±5 g) was defined as the percentage of the weight before and after cooking.


(1) $$\begin{array}{l} \text{Water holding capacity }(\%)\\ =\frac{\text{Moisture content }(\text{g})-\text{Released water }(\text{g})}{\text{Moisture content }(\text{g})}\times 100 \end{array}$$

(2) $$\begin{array}{l} \text{Water contents }(\%)\\ =\frac{\text{Weight before drying }(\text{g})-\text{Weight after drying }(\text{g})}{\text{Weight before drying }(\text{g})}\times 100 \end{array}$$

### Color analysis

After 30 min of blooming, instrumental color measurement of the surface for meat was conducted using a spectrophotometer (CM-5; Konica Minolta Censing Inc., Tokyo, Japan). The colorimeter was calibrated using standard white and black plates before each measurement. The Commission Internationale de l’Eclairage (CIE) L* (lightness), a* (redness), and b* (yellowness) values were obtained from the average values of six readings on the surface of the loin samples. The hue angle (tan−1 [b*/a*]) and chroma ([a*2+b*2]1/2) were calculated using the CIE L*, a*, and b*.

### Far-infrared ray emissivity and radiant energy

To investigate the FIR properties of OR and OS, samples were prepared with a specific size (30 mm width×30 mm length×5 mm height). An FT-IR spectrometer was used over the range of 5 to 20 *μ*m at 37°C according to the standard measurement method (KFIA-FI-1005) provided by the Korea Far Infrared Association.

### Scanning electron microscopy

Scanning electron microscopy was performed according to the method described by Shin et al [12] with a few modifications. Broken pieces (1 mm width×1 mm length×1 mm height) from each *onggi* were placed carefully on aluminum stubs with carbon tape and coated with platinum under a vacuum for surface visualization. Samples were obtained using a field-emission scanning electron microscope (Carl Zeiss Microscopy, Thornwood, NY, USA).

### Quantification of immobilized *L. sakei* on *onggi*

*Lactobacillus* species in meat aged in OS were identified as *L. sakei* (Table 1). To analyze the *L. sakei* immobilization, counts of *Lactobacillus* spp. on the inner surface of *onggi*es were conducted via the swabbing method. The inner surface of *onggi* was gently swabbed with cotton swabs soaked in 0.85% sodium chloride with 0.1% Tween-20. The swab head was immersed in 500 μL of sterile saline solution for shaking out the bacteria, followed by centrifugation at 5,600×*g* for 10 min to collect the bacterial pellet. The collected pellet was then suspended in 200 μL of sterile saline solution and then spread on *lactobacilli* MRS broth with agar (Difco Laboratories, USA). The plates were then incubated at 37°C for 96 h.

**Figure 2:**
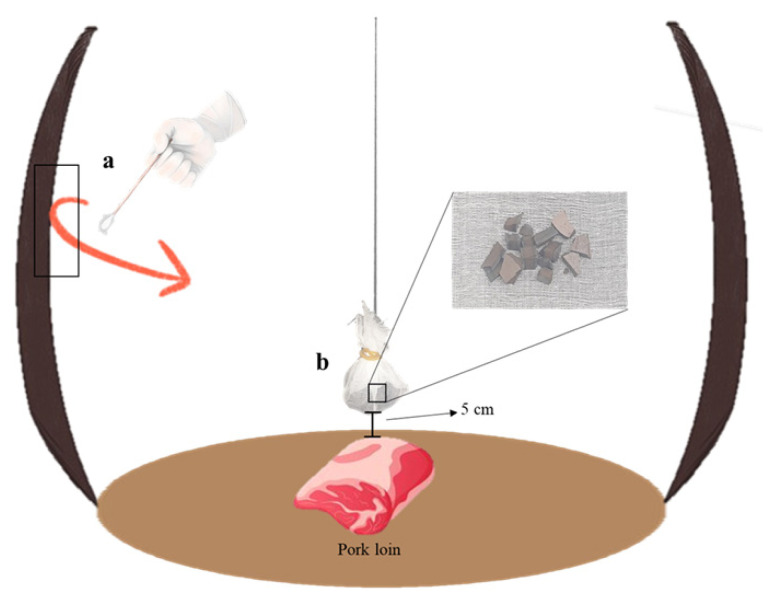
Schematic representation for collection of immobilized *L. sakei*. (a) Swabbing method using sterilized cotton swab. (b) Hung broken pieces to check the presence of *L. sakei* on cross-section of *onggi*.

To check for the presence of *L. sakei* on the cross-section of *onggi*es, a pure cotton gauze (15 cm width×15 cm length) was cut. Broken pieces (1 mm width×1 mm length×1 mm height) from each *onggi* were wrapped with cut cotton gauze and hung by silk thread during aging in *onggi* (Figure 2). The hung *onggi* pieces and the pork loin in *onggi* were placed 5 cm apart. On day 21, the hung pieces were blended with 0.85% saline solution for 2 min using a laboratory stomacher (Interscience Ind., France) to separate *L. sakei* on the cross-section. One milliliter of each dilution was poured into *lactobacilli* MRS broth with agar, followed by incubation at 37°C for 96 h.

### Microstructural properties

The pore diameter, total pore area, porosity, and density of OR and OS, which are important factors controlling the barrier properties, were measured using a mercury porosimeter (AutoPore IV9500; Micromeritics, Norcross, GA, USA). Broken pieces (5 mm width×5 mm length×5 mm height) from each *onggi* were used as the samples. Before intruding mercury in stepwise pressure increments in the range of 0.44 to 59858.85 psia, the undisturbed pieces with open pore structures were degassed in a vacuum under pressure. The average pore diameter was obtained by assuming that all pores were right cylinders; thus, when the volume (V = πr^2^L) was divided by the pore area (A = 2πrL), the average pore radius (r) was equal to 2 V/A. Porosity was derived from the ratio of the total intruded volume of mercury at the highest pressure determined (59,858.85 psia) to the total volume of the sample.

### Statistical analysis

All experiments were performed in triplicate. A general linear model was used to perform the analysis using SAS (version 9.4; SAS Institute Inc., Cary, NC, USA), and the results were reported as the mean±standard error of mean. After each analysis of variance, significant differences among means were further tested using the Student–Newman–Keuls test.

## RESULTS AND DISCUSSION

### Microbial analysis

Meat aged in VP had the lowest TPC and number of *Lactobacillus* spp. among the treatments during storage (Table 2). This was because the meat aged in VP was isolated from the external atmosphere using VP. Hulánková et al [13] mentioned that plastic bags for VP are effective gas barriers with sealing characteristics.

On day 21, meat aged in OS displayed the highest number of *Lactobacillus* spp. compared to those of the other methods, which may be due to the alteration of atmospheric composition in *onggi*. The growth of *Lactobacillus* spp. could be different according to remaining O_2_ contents in treatment. Meat aged in VP showed the lowest numbers of *Lactobacillus* spp. among all treatments due to their absolute anaerobic condition. It was supported by the fact that *L. sakei*, which belongs to facultative anaerobic bacteria, can grow slowly under O_2_-deficient condition but grows best with the presence of O_2_ [14]. Chung et al [4] showed that during soy sauce fermentation in *onggi*, the atmosphere with comparatively low O_2_ concentration activated the growth of lactic acid bacteria (LAB) in soy sauce after two months. Another finding also indicated that the early aerobic fermentation conditions could be changed to the relative anaerobic state during the fermentation of *kimchi* using *onggi* [15]. The authors stated that this comparative anaerobic condition increased the ratio of anaerobic bacteria such as LAB. Therefore, the environment in OS on day 21 may have changed to a relatively anaerobic condition compared to that in OR on day 21, which would be beneficial for the growth of *Lactobacillus* spp.

The highest TPC was observed in meat aged in OR after more than seven days of storage, followed by those aged in OS and VP (p<0.05). The comparatively lower TPC of meat aged in OS compared to that in OR on day 21 was also possibly due to the lower O_2_ concentration in OS along with the antibacterial activity of *Lactobacillus* spp. Low O_2_ concentration in OS could have also caused a lower aerobic bacterial count at day 21. The ratio of *Lactobacillus* spp. to TPC displayed similar values between meat aged in OR and OS, except for those aged in OS on day 21 (70.04%) (Table 2). The increased number of *Lactobacillus* spp. in meat aged in OS on day 21 lowered the activity of other bacteria, which agrees with da Costa et al [16]. The authors demonstrated that *Lactobacillus* spp. can excrete substances with antimicrobial properties, such as bacteriocins and organic acids.

A high number of *Lactobacillus* spp. was identified in meat aged in OS, especially on day 21 (Table 2). Because the counts of LAB were very low (about 1 log CFU/g) in dry-aged beef on 21 days of storage [13], it may be meaningful to study *Lactobacillus* spp. in meat aged in OS. In the present study, the *Lactobacillus* spp. in meat aged in OS were identified as *Lactobacillus sakei* (Table 1). *L. sakei* produces lactic acid as the sole or main product of carbohydrate metabolism [17].

### pH and volatile basic nitrogen

The pH and VBN of meat on day 21 were the highest for meat aged in OR, followed by those of meat aged in OS and VP (Table 3), which was the same trend as that of TPC (Table 2). The pH and VBN values of meat aged in VP were stable during the length of storage, whereas those of meat aged in OR and OS significantly increased on day 21. pH and VBN are the most definitive spoilage indicators of fresh meat. In Korea, the limitation of pH and VBN of meat for sale are 6.2 and 20 mg/100 g, respectively; meat with values beyond these parameters are regarded as spoiled [10,18]. Based on this information, meat aged in OS on day 21 (pH = 6.2 and VBN = 20 mg/100 g) may be acceptable to consumers, while not so for meat aged in OR on day 21.

Meat aged in VP displayed the lowest pH (p>0.05) and VBN values (p<0.05) among all treatments on day 21. Microbial growth affects both the pH and VBN; the amounts of basic products derived from microorganism proteolysis can increase the pH and VBN content in meat [10]. Lee [2] explained that the lower pH in wet-aged meat compared to that in dry-aged meat was attributed to the high production of amine and ammonia in dry-aged meat and/or the decrease in pH in wet-aged meat due to the growth of LAB. In the same manner, the lower pH and VBN values of meat aged in OS compared to those of meat in OR on day 21 could be due to the lower amount of amine and ammonia in meat aged in OS on day 21, or increased *L. sakei* in meat aged in OS on day 21. These results are consistent with those of Zhang et al [19], who reported that *L. sakei* reduced the generation of VBN. *L. sakei* produces fewer basic compounds than other species of microorganisms because of its non-aminogenic and decarboxylase-negative properties [20]. L. sakei is effective in reducing amine accumulation, with a reduction of up to 91% and 74% for putrescine and cadaverine, respectively, which are associated with the activity of food-borne pathogenic bacteria [20]. Latorre-Moratalla et al [20] also found that decarboxylase-negative *L. sakei* induced much lower amounts of amines, such as tyramine, cadavenine, and phenylethylamine, than decarboxylase-positive *Lactobacillus curvatus*.

### 2-thiobarbituric acid reactive substances

TBARS values of meats aged in OR and OS were higher than those in VP during the aging period (p<0.05; Table 3). With respect to 21 days of storage, the values of meats aged in OR and OS were not significantly different. However, all treatments during the aging period could be acceptable due to their values being less than 2 mg MDA/kg which is considered the threshold for rancid off-flavors [1].

Lower TBARS values for meat aged in VP than for those aged in OR and OS could be attributed to less O_2_ exposure in meat aged in VP. Because meats aged in VP were vacuum-packed, they were less exposed to O_2_ than those aged in OR and OS. Indeed, Zakrys et al [21] showed that lipid oxidation is positively correlated with O_2_ concentration during storage.

The meat aged in OS displayed a similar TBARS value to that aged in OR on day 21 (Table 3), possibly due to the aforementioned low O_2_ concentration in OS on day 21 and the high level of *L. sakei* on meat aged in OS on day 21. The lower O_2_ concentration in OS on day 21 delayed lipid oxidation. In contrast, high lactic acid levels generated from *Lactobacillus* spp. may accelerate lipid oxidation [22]. Increased H^+^ concentration by lactic acid can generate a highly reactive radical, hydroperoxyl radical (HO_2_), and stimulate the dissociation of protein-bound iron, promoting free radical generation [22]. Thus, the decrease in lipid oxidation from low O_2_ concentration was most likely offset by the increase in lipid oxidation from the high amount of lactic acid in meat aged in OS on day 21.

### Water activity

**Figure 3:**
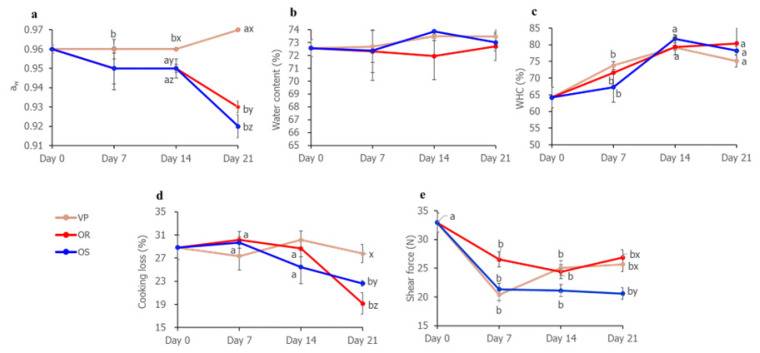
Shear force and water-related quality properties of meat aged using different methods. (a) Water activity (a_w_). (b) Water content (%). (c) Water holding capacity (WHC). (d) Cooking loss (%). (e) Shear force (N) of pork loins. VP/OR/OS refers to vacuum-packaging (VP), *onggi* containing red clay only (OR), and *onggi* containing 30% red clay and 70% scoria powder (OS). ^a,b^ Means within the same treatment with different superscripts differ significantly (p<0.05). ^x–z^ Means within the same aging period with different superscripts differ significantly (p<0.05).

Meat aged in OS had a significantly lower water activity (a_w_) value than meats aged in OR and VP on day 21 (p<0.05; Figure 3a). The lower a_w_ of meat aged in OS would be caused by the increased number of hydrolyzed proteins due to the higher the number of *L. sakei* on meat aged in OS on day 21. In several studies [23,24], more water was stabilized or held by hydrophilic groups on increased proteins amounts, such as small peptides and amino acids, from proteolysis of *L. sakei*. It might induce formation of much bound water that microorganisms cannot use for their growth [25]. Therefore, low aw in meat aged in OS on day 21 was probably attributed to increase of bound water from small peptide and amino acids via proteolytic activity of *L. sakei*.

### Water content and water-holding capacity

Water content displayed insignificant differences among all treatments during aging period (Figure 3b). Lee et al [26] reported that *onggi* with lower water permeability did not make a difference on water loss of products in *onggi* during fermentation of 3 month. Same to the previous study, the different permeability between OR and OS could have not influenced water content for 21 day.

WHC of meats aged in VP, OR, and OS showed an increasing tendency over the aging period (Figure 3c). There was increase of WHC in muscle through swelling of muscle fibers during aging [27]. Similar WHC results between meats aged in OR and in OS on day 21 (Figure 3e) were attributable to aforementioned increased bound water and lower pH of meat aged in OS than in OR on day 21. Increased bound water by proteolysis of *L. sakei* on meat aged in OS on day 21 might increase WHC [23]. However, pH (5.85) on meat aged in OS on day 21, which was nearer at isoelectric point than that in OR on day 21, could cause lower WHC. Kang et al [14] stated that pH, which is nearer to isoelectric point, decreases WHC. The compensation effect between increased bound water and pH of meat aged in OS on day 21 would affect the result in WHC of meats aged in OS and OR, showing no significant difference between them.

### Cooking loss and shear force

Cooking loss for meat aged in VP displayed the highest value, followed by those aged in OS and OR on day 21 (Figure 3d). Another report also found that the high cooking loss of wet aged beef occurred due to vacuum pressure [1]. The higher cooking loss on meat aged in OS than that in OR at day 21 could be attributed to the high proteolytic property from *L. sakei*. Purslow et al [27] mentioned that proteolysis produced protein fragments which are more easily lost from protein structure with water during cooking. In addition, sarcoplasmic proteins have an influence on networked linkage each other or with myofibrillar proteins, followed by trapping more water in the structure [28]. Stability reduction of myofilaments and sarcoplasmic proteins by proteolysis incurs greater protein denaturation by cooking [27].

The shear force (N) of meat aged in OS was lower than that of meat aged in OR and VP on day 21 (Figure 3e). The lower shear force (N) on meat aged in OS than those in OR and VP on day 21 would also be owing to *L. sakei*. Meat tenderization is attributed to activity of proteolytic enzymes in animal muscle (i.e. caspase, proteasome, cathepsins, calpains) or/and from microorganisms [2]. Low pH and aw, induced by a number of *Lactobacillus* species, can activate endogenous proteinase in meat [29,30]. Proteolytic enzymes from *Lactobacillus* spp. [31] also might tenderize muscle structure.

### Color analysis

**Figure 4:**
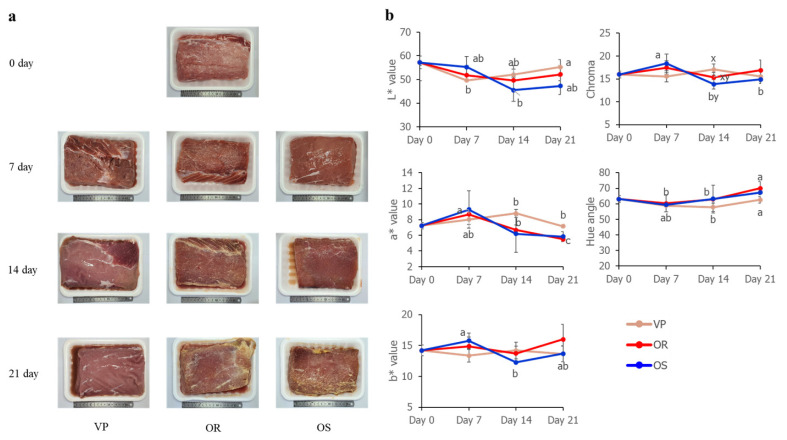
Change in color of meat aged using different methods. (a) Visual appearance. (b) Color changes of surface for pork loin by CIE value. VP/OR/OS refers to vacuum-packaging (VP), *onggi* containing red clay only (OR), and *onggi* containing 30% red clay and 70% scoria powder (OS). ^a–c^ Means within the same treatment with different superscripts differ significantly (p<0.05). ^x,y^ Means within the same aging period with different superscripts differ significantly (p<0.05).

As shown in Figure 4a, a yellow appearance was seen, which is consistent with the results of Purslow et al [27], where a yellow discoloration by LAB on prepackaged refrigerated German ‘Weisswurst’ was apparent. However, in the present study, the color of the surface for meat was not significantly different among treatments on the same storage day (Figure 4b).

### Far-infrared ray emissivity and radiant energy

FIR promotes meat aging and sterilizes pathogens [8]. Lin [9] found a lower pH, TPC, and VBN when FIR-irradiating ceramic powder was added for packaging at 4°C, and assumed that FIR of ceramic powder had bacteriostatic effects. In addition, *L. sakei* was supposed to have different sensitivities to FIR compared to that of other bacteria due to the ability of LAB to survive under conditions of environmental stress [32]. Thus, in the present study, the emissivity and radiant energy of FIR were measured for OR and OS to elucidate the reasons for the higher number of *L. sakei* and lower TPC in meat aged in OS compared to that aged in OR on day 21.

Table 4 displays similar emissivity (0.9080 and 0.9065) and radiant energy (3.500×10^2^ and 3.495×10^2^ w/m^2^) between OR and OS at 37°C, respectively, which is the minimal temperature for analysis using a commercial emissivity detector (KFIA-FI-1005). The values needed to be considered as those at 4°C, which was the aging condition used in the current study. The FIR emissivity is the ratio of the FIR radiant emittance of an object to that of a black body and the radiant emittance is the radiated energy per unit area of the object’s surface. According to Plan’s law, the wavelength of the radiated energy with the highest intensity is inversely proportional to absolute temperature [7]. Therefore, because the emissivity of OR and OS may be equally affected by the shifts of the emittance spectrum peak to longer wavelengths according to the decrement of the absolute temperature based on Plan’s law, the emissivity values for OR and OS would show similar values between 37°C and 4°C.

The difference in radiant energy at 4°C and 37°C can be predicted using the Stefan–Boltzmann law; radiant energy represents the energy density of the FIR. That is, a higher energy density denotes a higher FIR irradiation per unit area. The radiant energy is calculated according to the Stefan–Boltzmann law (3) as follows [7,33]:


(3) $$\text{P}=\psi \;{\text{abT}}^{4}\;(\text{w}/{\text{m}}^{2})$$

where P is the radiant energy, ψ is the emissivity, a is the Stefan-Boltzmann constant (5.7×10^−8^ W/m^2^·k^4^, b is the surface area of the emitter (m^2^), and T is the absolute temperature (K). Based on this law, only ψ (emissivity) and T (absolute temperature) would differ between 4°C and 37°C. That is, the radiant energies of OR and OS would decrease equally in line with the decrement of the emissivity and the four squares of the absolute temperature from those at 37°C and 4°C. Thus, the radiant energies of the OR and OS at 4°C were probably similar to those at 37°C.

In addition, the same emissivity and radiant energy of OR and OS could be attributed to a similar constitution ratio of metal oxides. The composition ratio of main components that compose red clay was 40% to 50% SiO_2_, 20% to 30% Al_2_O_3_, 5% to 6% FeO_3_ [34], and that of scoria was 30% to 50% SiO_2_, 10% to 20% Al_2_O_3_, and 10% to 20% FeO_3_ [5].

In the present study, OR and OS radiated high FIR, which was similar to previous studies where both red clay and scoria radiated high FIR at high temperatures [5,35]. However, these results could be limited because of the aging conditions for low temperature and dark sites. According to Park [35], absorbable energy sources, such as high temperatures or large amounts of strong light, must exist for ceramics to radiate a high FIR. Thus, ceramics at low temperatures (4°C) or in dark sites radiate less FIR because there is a small energy source for ceramics to absorb. Therefore, it was assumed that FIR in OR and OS was a minor factor affecting the number of *L. sakei* on meat aged in OS on day 21.

### Quantification of immobilized *Lactobacillus sakei* on *onggi*

According to Seo et al [3], an increase in microbial load can alter the atmospheric composition of *onggi*. Since *Lactobacillus* spp. are classified as airborne microorganisms [32], there is a possibility for *L. sakei* in pork loin to move to *onggi*. Therefore, differences in the counts of immobilized *L. sakei* on the surface and cross-section of OR and OS were evaluated (Figure 2).

**Figure 5:**
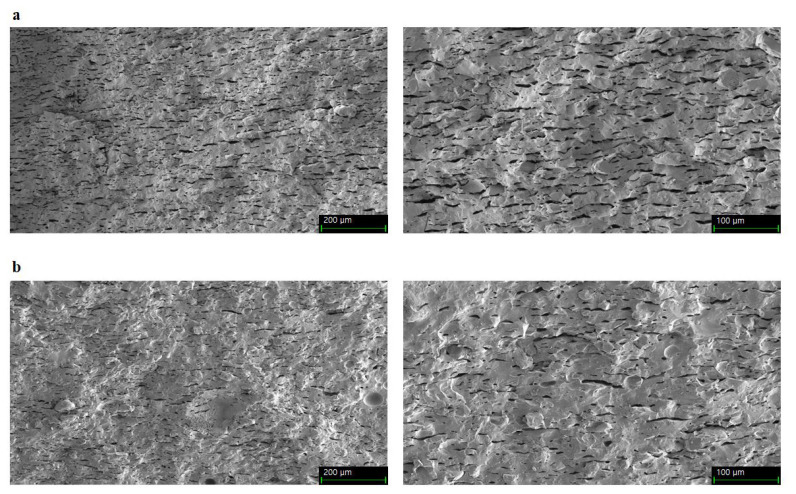
Scanning electron micrographs of the earthenware surface. (a) Inner cross-section of OR (×250, left side; ×500, right side). (b) Inner cross-section of OS (×250, left side; ×500, right side). OR/OS refers to *onggi* containing red clay only (OR) and *onggi* containing 30% red clay and 70% scoria powder (OS).

Immobilization can be affected by the pore size and surface characteristics of the porous material and the attached proteins [36]. Figure 5 shows a larger pore size in OR than in OS. Different pore sizes between OR and OS may create different immobilizations of *L. sakei* on OR and OS. Pores on the surface of *onggi* provide microhabitats where LAB can proliferate [15], and pore size relative to protein size influences protein immobilization [36]. Liu et al [37] found that an increase in the pore size up to 30 nm rendered enzyme immobilization on porous sieves more suitable. In porous ceramics for microbe immobilization, the pore size, which is 10 times longer than the microbe size, is stable for microbial habitats [38].

*L. sakei* may have a high affinity for OR and OS. Indeed, ceramic materials such as *onggi* have remarkable affinity for microorganisms [38], and *Lactobacillus* spp. were successfully immobilized in a shell composed mainly of SiO_2_, the major ingredient of scoria [39]. The surface charge of porous SiO_2_ are complementary to proteins such as surface proteins of *Lactobacillus* spp.; that is, electrostatic interactions between porous SiO_2_ and proteins influence immobilization [36]. Therefore, similar SiO_2_ contents in OR and OS would not affect the difference in *L. sakei* immobilization.

However, contrary to expectations in the current study, *L. sakei* was not detected on any inner surface or cross-section of OR and OS (data not shown), which meant *L. sakei* immobilization could not be confirmed. This may be because *L. sakei* from pork loins did not move to *onggi*es through the air for immobilization to take place. Thus, *L. sakei* immobilization may not be related to the results of meat aged in OS on day 21.

### Microstructural properties

Microstructural properties, such as pore diameter, porosity, and permeability, of *onggi* can control the growth of microorganisms on the food products in *onggi* [4,35]. Porosity implies the amounts of openings within a material [4]. In the current study, OS had higher values for total pore area and a smaller pore diameter than OR (Table 5). This means that OS had a larger number of smaller pores, which induced a higher total pore area compared to OR. Different microstructural properties of OS compared to those of OR could affect differently to the growth of microbes on the food products in *onggi*.

Several studies have shown that *onggi* having a smaller pore size promotes the growth of LAB in food. When kimchi, soy sauce, and grapes were fermented in *onggi*, the *onggi* with a smaller pore size caused lower O_2_ and higher CO_2_ concentrations, followed by an increase in the growth of LAB in stored foods [3,4,15]. The composition of gases in *onggi* can change depending on the interplay between the product respiration rate and material permeability characteristics [40]. The respiration of a product decreases O_2_ and increases CO_2_ concentrations in its container [41]. The permeability of *onggi* allowed the outer sufficient O_2_ to pass through the *onggi*, avoiding anaerobic respiration, and the inner excessive CO_2_ to permeate the *onggi* to prevent physiological disorders [3,41]. In addition, the water permeability of *onggi* helps to avoid moisture saturation and maintain appropriate relative humidity for storing fresh products [3]. Therefore, the gas and water permeability of *onggi* are essential criteria for an appropriate modified atmosphere for the food products within [40].

**Figure 6:**
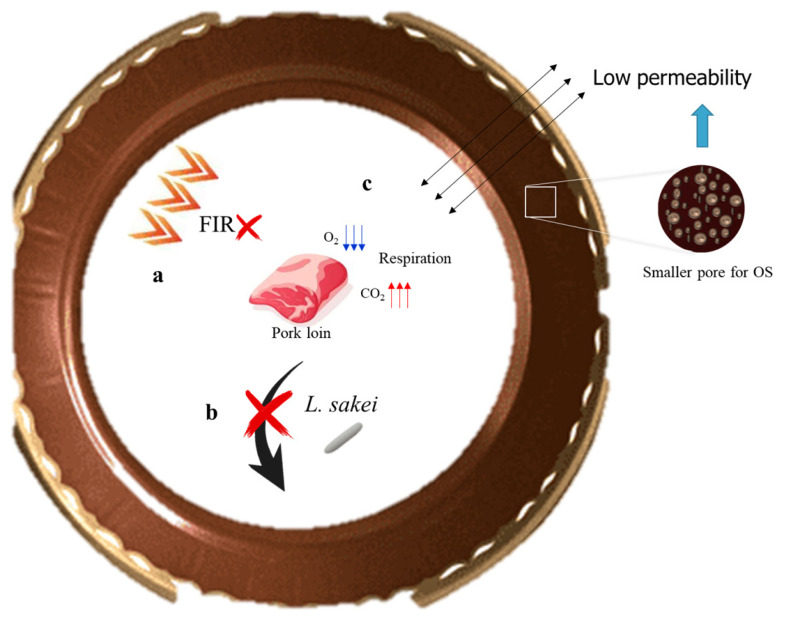
Schematic representation of mechanism for high number of *L. sakei* on meat aged in OS on day 21. (a) Far-infrared ray (FIR) irradiation. (b) Quantification of immobilized *L. sakei*. (c) Change of atmospheric composition according to low permeability of *onggi* and respiration of stored products.

In the present study, it was assumed that a smaller pore size in OS induced lower permeability, increasing the number of LAB in pork loins. The permeability of a material is expressed as a function of porosity and mean pore diameter. A previous study explained an equation to calculate permeability as follows (4) [42]:


(4) $$\psi ={\text{pd}}^{2}/16{{\text{f}}_{\text{CK}\tau }}^{2}$$

where ψ is the Darcian permeability, p is the efficient porosity, d is the mean pore diameter, f_CK_ is the Carman-Kozeny coefficient, and τ is the tortuosity of the pore channels, ratio between the fluid-traveled mean length through the porous medium, and thickness of the porous medium. This permeability equation (4) can elucidate the discrepancy in permeability relative to OR and OS. When the pores become smaller, the tortuosity increases. In this case, porosity could be negligible because it had little effect on permeability for pore diameters shorter than 6 μm [42]. Consequently, the permeability of OS was directly proportional to the square of the mean pore diameter and inversely proportional to the square of the tortuosity. It was suggested that OS had a smaller mean pore diameter and higher tortuosity than that of OR. Thus, the permeability of OS was much lower than that of OR. Figure 6 shows a schematic diagram of the mechanism of the high number of *L. sakei* on meat aged in OS on day 21.

## CONCLUSION

*Onggi* supplemented with scoria powder improved the meat quality of pork loin by increasing the number of *L. sakei*. On day 21, the meat aged in OS exhibited lower pH, shear force, VBN, and a_w_ than those of meat aged in OR on day 21, along with an increase in the number of *L. sakei*. This microorganism accounted for 6.8 log CFU/g in 9.72 log CFU/g TPC in meat aged in OS on day 21. FIR and *L. sakei* immobilization rarely affected the difference between OR and OS. Rather, the lower permeability of OS compared to OR may create a favorable environment for *L. sakei* proliferation. Therefore, *onggi* containing scoria powder improved the quality and microbiological safety of dry-aged pork loins. The findings of the present study suggest a novel meat aging process with a high number of *L. sakei*. Sensory evaluation, the optimum ratio of scoria, and glazing for *onggi* should be investigated in future studies.

## Figures and Tables

**Table 1:** BlastN results used to identify *Lactobacillus* species from aged meat on day 14 and 21

Source of isolation	Top hit	NCBI accession n.	E value	Identity (%)	Query cover (%)
Day 14	*Lactobacillus sakei* subsp. sakei gene for 16S ribosomal RNA, partial	LC064899.1	0.0	99	99
Day 14			0.0	99	98
Day 21			0.0	99	98
Day 21			0.0	100	98

**Table 2:** Microbial analysis for meat aged using different methods

Traits	Treatment	Storage day				SEM

		0	7	14	21	
TPC (log CFU/g)	VP	2.85^c^	2.98^cz^	4.52^bz^	5.00^az^	0.111
	OR	2.85^d^	6.33^cx^	8.22^bx^	9.95^ax^	0.075
	OS	2.85^d^	5.38^cy^	7.55^by^	9.71^ay^	0.088
	SEM	-	0.080	0.159	0.053	-
Lactobacillus spp. (log CFU/g)	VP	nd	1.79^by^	2.63^by^	4.34^az^	0.401
	OR	nd	3.52^cx^	3.97^bx^	4.99^ay^	0.129
	OS	nd	2.80^cxy^	3.62b^xy^	6.80^ax^	0.164
	SEM		0.307	0.299	0.146	-
The ratio of *Lactobacillus* spp./TPC (%)	VP	0	60.07	58.19	86.80^x^	11.117
	OR	0	55.61	48.30	50.15^z^	1.756
	OS	0	52.05^b^	47.95^b^	70.04^ay^	2.581
	SEM	-	9.344	6.399	2.253	-

SEM, standard error of the mean; TPC, total plate count; VP, vacuum packaging; OR, *onggi* containing red clay only; OS, *onggi* containing 30% red clay and 70% scoria powder; nd, not detected.

a–d Means within the same row with different superscripts differ significantly (p<0.05).

x–z Means within the same column with different superscripts differ significantly (p<0.05).

**Table 3:** pH, volatile basic nitrogen, and 2-thiobarbituric acid reactive substances for meat aged using different methods

Traits	Treatment	Storage day				SEM

		0	7	14	21	
pH	VP	5.55	5.77	5.56	5.70^y^	0.092
	OR	5.55^b^	5.80^b^	5.94^b^	6.45^ax^	0.116
	OS	5.55^b^	5.62^b^	5.64^b^	5.85^ay^	0.056
	SEM	0.007	0.095	0.121	0.098	-
VBN (mg/100 g)	VP	7.82	8.75^y^	8.16^y^	8.75^z^	0.280
	OR	7.82^d^	10.57^cx^	13.3^bx^	25.55^ax^	0.276
	OS	7.82^b^	8.63^by^	10.38^bxy^	16.57^ay^	1.158
	SEM	0.309	0.296	0.894	1.006	-
TBARS (mg MDA/kg)	VP	0.12^b^	0.20^ay^	0.18^ay^	0.17^ay^	0.012
	OR	0.12^b^	0.41^ax^	0.34^ax^	0.34^ax^	0.046
	OS	0.12^b^	0.34^axy^	0.40^ax^	0.28a^xy^	0.041
	SEM	0.008	0.044	0.043	0.037	-

SEM, standard error of the mean; VP, vacuum packaging; OR, *onggi* containing red clay only; OS, *onggi* containing 30% red clay and 70% scoria powder; VBN, volatile basic nitrogen; TBARS, 2-thiobarbituric acid reactive substances; MDA, malonaldehyde.

a–d Means within the same row with different superscripts differ significantly (p<0.05).

x–z Means within the same column with different superscripts differ significantly (p<0.05).

**Table 4:** Far-infrared irradiation for different *onggi*es

Treatment	Emissivity (5 to 20 μm)	Radiant energy (W/m^2^·μm, 37°C)
OR	0.9080	3.500×10^2^
OS	0.9065	3.495×10^2^
SEM	0.000	0.354

OR, *onggi* containing red clay only; OS, *onggi* containing 30% red clay and 70% scoria powder; SEM, standard error of the mean.

**Table 5:** Microstructural properties for different *onggi*es

Treatment	Pore diameter (nm)	Total pore area (m^2^/g)	Porosity (%)	Density (g/mL)
OR	258.93^x^	0.54^y^	8.09	2.52
OS	172.23^y^	0.77^x^	7.65	2.50
SEM	5.559	0.022	0.163	0.005

OR, *onggi* containing red clay only; OS, *onggi* containing 30% red clay and 70% scoria powder; SEM, standard error of the mean.

x,y Means within the same column with different superscripts differ significantly (p<0.05).

## References

[1] Ha M, McGilchrist P, Polkinghorne R (2019). Effects of different ageing methods on colour, yield, oxidation and sensory qualities of Australian beef loins consumed in Australia and Japan. Food Res Int.

[2] Lee HJ (2018). Factors affecting flavor of dry-aged beef and mechanism for the flavor development [dissertation].

[3] Seo GH, Chung SK, An DS, Lee DS (2005). Permeabilities of Korean earthenware containers and their potential for packaging fresh produce. Food Sci Biotechnol.

[4] Chung SK, Lee KS, Lee DS (2006). Fermentation of Kanjang, Korean soy sauce, in porosity-controlled earthenwares with changing the mixing ratio of raw soils. Korean J Food Sci Technol.

[5] Kang H (2018). Effect of mixture composed of Jeju’ scoria and Ecklonia cava on anti-inflammation. Biomed Sci Lett.

[6] Cho JO, Lee HW, Mok YS (2014). Sterilization of scoria powder by corona discharge plasma. Appl Chem Eng.

[7] Choi TS (2001). Application of infrared rays radion packaging material resource. The Monthly Packaging World.

[8] KIPO (2019). Container for meat aging [Internet].

[9] Lin LC (2003). The new storage technology: effect of far infrared ray (FIR) ceramic sheet package on storage quality of pork loin. Asian-Australas J Anim Sci.

[10] Lee HJ, Choe J, Yoon JW (2018). Determination of salable shelf-life for wrap-packaged dry-aged beef during cold storage. Korean J Food Sci Anim Resour.

[11] Kim SS, Lee YE, Kim CH, Min JS, Yim DG, Jo C (2022). Determining the optimal cooking time for cooking loss, shear force, and off-odor reduction of pork large intestines. Food Sci Anim Resour.

[12] Shin DJ, Lee HJ, Lee D, Jo C, Choe J (2020). Fat replacement in chicken sausages manufactured with broiler and old laying hens by different vegetable oils. Poult Sci.

[13] Hulánková R, Kameník J, Saláková A, Závodský D, Borilova G (2018). The effect of dry aging on instrumental, chemical and microbiological parameters of organic beef loin muscle. LWT.

[14] Kang SN, Kim IS, Kim HY (2018). Meat Science.

[15] Han KI, Kim MJ, Kwon HJ, Kim YH, Kim WJ, Han MD (2013). The effect of container types on the growth of bacteria during kimchi fermentation. Korean J Food Nutr.

[16] da Costa WKA, de Souza GT, Brandão LR (2018). Exploiting antagonistic activity of fruit-derived Lactobacillus to control pathogenic bacteria in fresh cheese and chicken meat. Food Res Int.

[17] Tannock GW (2004). A special fondness for lactobacilli. Appl Environ Microbiol.

[18] Kim HJ, Kim HJ, Kim KW (2022). Effect of feeding alfalfa and concentrate on meat quality and bioactive compounds in Korean native black goat loin during storage at 4°C. Food Sci Anim Resour.

[19] Zhang Y, Zhu L, Dong P (2018). Bio-protective potential of lactic acid bacteria: Effect of Lactobacillus sakei and Lactobacillus curvatus on changes of the microbial community in vacuum-packaged chilled beef. Asian-Australas J Anim Sci.

[20] Latorre-Moratalla ML, Bover-Cid S, Bosch-Fusté J, Veciana-Nogués MT, Vidal-Carou MC (2014). Amino acid availability as an influential factor on the biogenic amine formation in dry fermented sausages. Food Control.

[21] Zakrys PI, Hogan SA, O’sullivan MG, Allen P, Kerry JP (2008). Effects of oxygen concentration on the sensory evaluation and quality indicators of beef muscle packed under modified atmosphere. Meat Sci.

[22] Groussard C, Morel I, Chevanne M, Monnier M, Cillard J, Delamarche A (2000). Free radical scavenging and antioxidant effects of lactate ion: an in vitro study. J Appl Physiol.

[23] Law BA, Kolstad J (1983). Proteolytic systems in lactic acid bacteria. Antonie van Leeuwenhoek.

[24] Schlesser JE, Schmidt SJ, Speckman R (1992). Characterization of chemical and physical changes in Camembert cheese during ripening. J Dairy Sci.

[25] Mermelstein NH (2009). Measuring moisture content and water activity. Food Technol.

[26] Lee KS, Lee YB, Lee DS, Chung SK (2006). Quality evaluation of Korean soy sauce fermented in Korean earthenware (Onggi) with different glazes. Int J Food Sci Technol.

[27] Purslow PP, Oiseth S, Hughes J, Warner RD (2016). The structural basis of cooking loss in beef: Variations with temperature and ageing. Food Res Int.

[28] Liu J, Puolanne E, Ertbjerg P A new hypothesis explaining the influence of sarcoplasmic proteins on the water-holding of myofibrils.

[29] Casaburi A, Di Monaco R, Cavella S, Toldrá F, Ercolini D, Villani F (2008). Proteolytic and lipolytic starter cultures and their effect on traditional fermented sausages ripening and sensory traits. Food Microbiol.

[30] Xiao Y, Liu Y, Chen C, Xie T, Li P (2020). Effect of Lactobacillus plantarum and Staphylococcus xylosus on flavour development and bacterial communities in Chinese dry fermented sausages. Food Res Int.

[31] Kieliszek M, Pobiega K, Piwowarek K, Kot AM (2021). Characteristics of the proteolytic enzymes produced by lactic acid bacteria. Molecules.

[32] Kongo Marcelino (2013). Lactic Acid Bacteria – R & D for Food, Health and Livestock Purposes.

[33] Ceramicx (2020). Far infrared ray: the law of heating [Internet].

[34] Lee KY, Lee HH, Ham SC (2019). A study on far-infrared radiation and proliferation of ocherous cotton quilt fabrics. J Naturop.

[35] Park MY (1991). Far infrared irradiation ceramic technology. Bull Food Technol.

[36] Lei J, Fan J, Yu C (2004). Immobilization of enzymes in mesoporous materials: controlling the entrance to nanospace. Microporous Mesoporous Mater.

[37] Liu Y, Xu Q, Feng X, Zhu JJ, Hou W (2007). Immobilization of hemoglobin on SBA-15 applied to the electrocatalytic reduction of H2O2. Anal Bioanal Chem.

[38] KIPO (2011). Porous ceramic carrier for microorganisms using sawdust and method for manufacturing the ceramic carrier [Internet].

[39] Zhao Z, Xie X, Wang Z (2016). Immobilization of Lactobacillus rhamnosus in mesoporous silica-based material: an efficiency continuous cell-recycle fermentation system for lactic acid production. J Biosci Bioeng.

[40] Hussein Z, Caleb OJ, Opara UL (2015). Perforation-mediated modified atmosphere packaging of fresh and minimally processed produce—a review. Food Packag Shelf Life.

[41] Yun JH, An DS, Lee KE, Jun BS, Lee DS (2006). Modified atmosphere packaging of fresh produce using microporous earthenware material. Packag Technol Sci.

[42] Wang B, Zhang H, Phuong HT, Jin F, Yang JF, Ishizaki K (2015). Gas permeability and adsorbability of the glass-bonded porous silicon carbide ceramics with controlled pore size. Ceram Int.

